# Enhanced Lithium Storage in Reduced Graphene Oxide-supported M-phase Vanadium(IV) Dioxide Nanoparticles

**DOI:** 10.1038/srep30202

**Published:** 2016-07-22

**Authors:** Hee Jo Song, Mingu Choi, Jae-Chan Kim, Sangbaek Park, Chan Woo Lee, Seong-Hyeon Hong, Byung-Kook Kim, Dong-Wan Kim

**Affiliations:** 1 Department of Materials Science and Engineering, Seoul National University, Seoul 151-744, Republic of Korea; 2 School of Civil, Environmental and Architectural Engineering, Korea University, Seoul 136-713, Republic of Korea; 3 High-Temperature Energy Materials Research Center, Korea Institute of Science and Technology, Seoul 136-791, Republic of Korea

## Abstract

Vanadium(IV) dioxide (VO_2_) has drawn attention as one of the most attractive electrode materials for lithium-ion batteries (LIBs), hence, much research has been conducted in various sectors in this field. However, to date, most of this research has focused on the VO_2_(B) polymorph, whereas electrochemical information on the use of VO_2_(M) in LIB electrodes is insufficient. Thus, it is worthwhile to explore the possibility of using VO_2_(M) for LIB electrode application, and to investigate whether its electrochemical properties can be improved. In this study, VO_2_(M) nanoparticles, incorporated with a reduced graphene oxide composite (NP-VO_2_/rGO), were successfully synthesized via a sol–gel assisted hydrothermal process by the chemical reduction of V_2_O_5_ gel, using hydrazine as the reducing agent. The particle size was less than 50 nm regardless of the presence of rGO. Also, NP-VO_2_/rGO exhibited a specific capacity of 283 mA h g^−1^ up to the 200^th^ cycle at a current density of 60 mA g^−1^, indicating its potential to be used in LIBs.

Rechargeable lithium-ion batteries (LIBs) have been developed in recent years as power sources in energy storage and conversion. From compact electronic equipment to electric vehicles and stationary energy storage systems, these batteries have been widely used because of their high power and energy density, together with long cycling life and good environmental compatibility^1^,^2^,^3^. Vanadium oxides (VO_x_), exist in various compositions (e.g., V_2_O_5_, V_3_O_7_, V_4_O_9_, V_6_O_13_, VO_2_, V_2_O_3_, etc.), depending on the oxidation state (5+ to 2+) of vanadium. They have attracted considerable attention as potential electrode materials for LIBs due to their low cost, high energy density, moderate work-potential, and the abundance of vanadium in nature^4^,^5^,^6^. Among them, vanadium(IV) dioxide (VO_2_) is mostly being tested for LIB cathode application. However, it also has a potential usage as an anode in LIB because it can be reacted with Li ions in the low-voltage ranges (0–3 V vs. Li/Li^+^).

It is well known that VO_2_ exhibits various polymorphic structures, namely, monoclinic VO_2_(M) with a distorted rutile structure that is thermodynamically stable, its metallic rutile structure VO_2_(R) that undergoes a phase transition at high-temperatures above 68 °C, thermodynamically metastable VO_2_(A) with a tetragonal structure, and metastable VO_2_(B) with a monoclinic structure^7^,^8^,^9^. Among these polymorphs, metastable VO_2_(B) has been the most widely researched for its potential in LIB application, due to its high specific capacity and its bilayered structure with edge-sharing VO_6_ octahedra that result in fast Li ion migration during insertion/desertion^1^,^4^,^9^. Conversely, few reports have addressed the electrochemical properties of stable VO_2_(M) for LIB application. This is due to the intrinsically low Li ion activity in its structure that originates from the corner-sharing VO_6_ octahedra, and yields a tunnel structure^10^,^11^. To date, Muñoz-Rojas *et al*.^10^ and Zhang *et al*.^12^ reported the electrochemical properties of hollow sphere VO_2_(M) and belt-like VO_2_(M), respectively, in non-aqueous electrolyte. Ni *et al*.^13^ estimated those of the VO_2_(M) nanoflower in aqueous electrolyte. In this research, however, electrochemical information was restricted and low specific capacities and cycling stabilities (lower than 100 mA h g^−1^ under the 50^th^ cycle) were obtained. Thus, it is worthwhile to investigate the possibility of using VO_2_(M) in LIB electrodes, and to discover ways to improve its electrochemical properties.

This study focuses on the utilization of VO_2_(M) as an electrode material for LIBs by developing a nanoscale VO_2_(M) powder below tens of nanometers in size. Furthermore, the improvement of its electrochemical properties was accomplished by using complex composites of conducting carbon media with VO_2_(M). By incorporating VO_2_(M) and conducting carbon, these composites could provide large contact areas between VO_2_(M) and the Li ion, together with fast charge transportation.

M-phase VO_2_ nanoparticles (denoted as NP-VO_2_) were synthesized via a facile sol-gel assisted hydrothermal process by chemical reduction of V_2_O_5_ gel using hydrazine reductant^14^,^15^. Although pristine graphene exhibits excellent electrical conductivity, it has poor processability due to its low solubility in many solvents. Graphene oxide (GO), on the other hand, has the advantage of easy access in aqueous solution^16^. Thus, the reduced graphene oxide (rGO) that is formed by the removal of the oxygen functional groups in GO, was used as a conducting carbon medium in order to improve the electronic conductivity and electrochemical properties of NP-VO_2_^17^,^18^. The VO_2_ NPs–rGO composite (denoted as NP-VO_2_/rGO) was also synthesized via a 1-step process with the chemical reduction of V_2_O_5_ gel and the colloidal dispersion of graphene oxide, simultaneously using more hydrazine reductant. Furthermore, this composite exhibited a higher capacity and a better cycling stability (over the 200^th^ cycle) than those of previous studies, and their electrochemical properties were investigated to determine their potential use in LIB electrodes.

## Results

### Preparation of VO_2_ NPs-rGO composite

**Figure 1:**
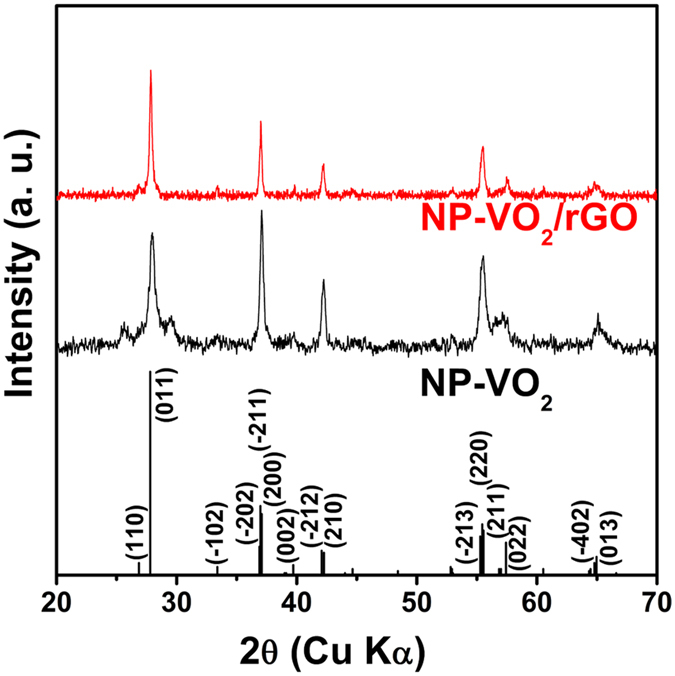
XRD analysis. XRD patterns of M-phase NP-VO_2_ and NP-VO_2_/rGO.

Prior to the synthesis of NP-VO_2_/rGO, pristine NP-VO_2_ was synthesized by the reduction of V_2_O_5_ gel using the strong reductant N_2_H_4_. The sol-gel was formed by dissolving V_2_O_5_ powder in H_2_O_2_. According to Ji *et al*., the amount of N_2_H_4_ was the critical factor in this NP-VO_2_ synthesis^15^. Thus, the products were synthesized by controlling the ratio of N_2_H_4_ to V_2_O_5_ gel. The lower graph in Fig. 1 shows the XRD pattern of NP-VO_2_ when additional 25% N_2_H_4_ (molar ratio of N_2_H_4_ to V_2_O_5_ is 0.625) was added. The reflection peaks in the pattern were in agreement with those of the VO_2_(M) reference data (JCPDS No. 43-1051; monoclinic unit cell with the space group P2_1_/C). Here, two small peaks at 2θ ~ 25.6° and ~29.6° in XRD pattern of NP-VO_2_ are related to 2θ = 27.8° (011) peak in VO_2_(M) phase, not related to impurity peaks. These peaks can be caused by excess or deficiency of reductant (N_2_H_4_) when hydrothermal reaction.

It is well-known that significant amounts of oxygen functional groups in GO are removed by chemical reduction using N_2_H_4_^19^. Therefore, it needs to put additional reductant in the GO-dispersed V_2_O_5_ gel prior to the hydrothermal reaction, in order to prepare NP-VO_2_/rGO. Shown in upper graph in Fig. 1, phase-pure and M-phase VO_2_-rGO could be synthesized by putting additional N_2_H_4_ in GO-dispersed V_2_O_5_ gel.

**Figure 2:**
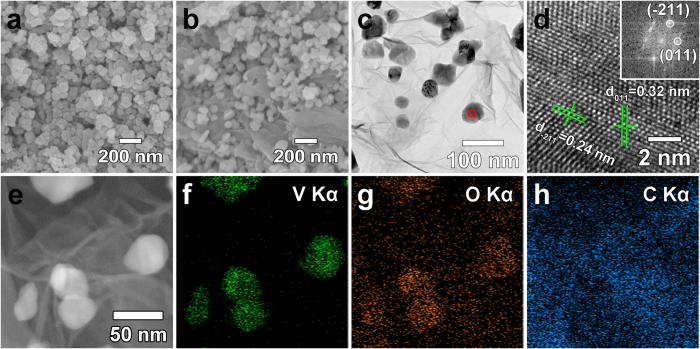
SEM and TEM characterization. (**a,b**) FESEM images of NP-VO_2_ and NP-VO_2_/rGO. (**c,d**) TEM image of NP-VO_2_/rGO and HR image obtained from the marked region in (**c**). The inset shows the reduced FFT patterns of the area in (**d**). (**e–h**) TEM EDS elemental mapping of an NP-VO_2_/rGO.

The synthesized particle morphologies and their size were examined using SEM and TEM analysis. Shown in Fig. 2a,b, respectively, both NP-VO_2_ and NP-VO_2_/rGO exhibited a nano spherical shape with a similar particle size. More detailed information on NP-VO_2_ and NP-VO_2_/rGO particles was determined by TEM analysis. Shown in Fig. 2c, VO_2_ particles with a size in the range of 20–50 nm, were well-dispersed in rGO. Also, they exhibited the same particle size as that of pristine NP-VO_2_ (see Supplementary Fig. S1), indicating that GO had little influence on the morphology and size during VO_2_ NPs formation. An HRTEM image (red-marked area in Fig. 2c) shows the clear lattice fringes in all the regions, indicating the high crystallinity of the VO_2_ NPs without an amorphous layer. The two lattice spacings—(011) and (−211) plane corresponding to lattice spacings of 0.32 nm and 0.24 nm, respectively—are consistent with the d-spacing of a VO_2_(M) structure. Further inspection of the reduced fast Fourier transform (FFT, inset of Fig. 2d) revealed that these are VO_2_(M) particles. The elemental composition of NP-VO_2_/rGO was determined via TEM EDS elemental mapping of V Kα, O Kα and C Kα (Fig. 2e–h). This identified that the particles are composed of V and O elements, while most of the C, together with a few O elements (considering that the O element in GO would be eliminated by the reductant as the reaction proceeded towards the formation of rGO), were detected in rGO.

**Figure 3:**
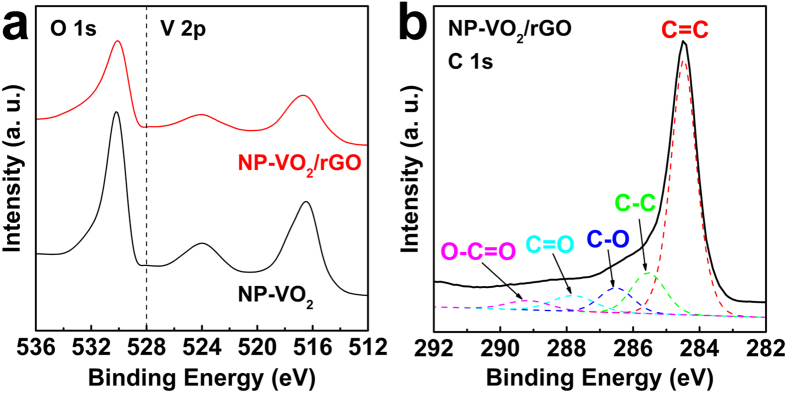
XPS analysis. (**a**) O 1s and V 2p level XPS spectra of NP-VO_2_ and NP-VO_2_/rGO. (**b**) C 1s level spectra of NP-VO_2_/rGO.

Figure 3a shows the XPS spectra of NP-VO_2_ and NP-VO_2_/rGO. Both composites exhibited the same peak positions of binding energy at the O 1s and V 2p levels. The obtained binding energy of the O 1s level is 530 eV. In the case of the V 2p level, spectra illustrate that the two peaks at 524.0 and 515.6 eV are attributed to the spin-orbit splitting of the V 2p_1/2_ and V 2p_3/2_, respectively. This corresponds to the characteristics of the V 4+ oxidation state^20^. Therefore, these results confirmed that VO_2_ NPs with an extra-pure phase could be synthesized, and also that GO had no influence on the valence state, as well as the morphology and size, during VO_2_ NPs formation. Figure 3b shows the C 1s level spectrum of NP-VO_2_/rGO. The five deconvolution peaks correspond to the C=C (284.5 eV), C–C (285.5 eV), C–O (hydroxyl, 286.6 eV), C=O (carbonyl, 287.8 eV), and O–C=O (carboxyl, 289.2 eV) groups, respectively. The C 1s level spectra of pristine GO exhibits strong peak intensities related to the oxygen functional groups^19^,^21^, whereas the carboxyl and carbonyl groups were significantly removed by the hydrazine reductant, leaving a small amount of hydroxyl groups in rGO, indicative of sufficient reduction of GO to rGO. The amount of rGO content included in the NP-VO_2_/rGO composite was estimated to be 12 wt% by TGA analysis (see Supplementary Fig. S2).

### NP-VO_2_/rGO for LIB electrode application

**Figure 4:**
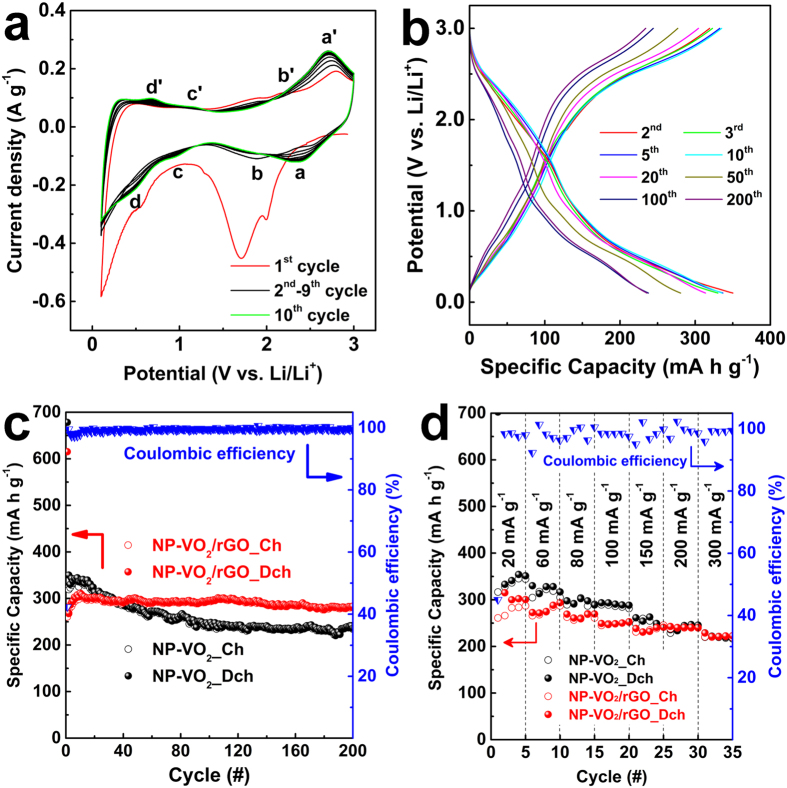
Electrochemical performance. (**a**) Cyclic voltammetry of NP-VO_2_ at a scan rate of 0.3 mV s^−1^ up to the 10^th^ cycle. (**b**) Galvanostatic voltage-specific capacity profiles of NP-VO_2_ at a scan rate of 60 mA g^−1^. (**c**) Specific capacities of NP-VO_2_ and NP-VO_2_/rGO at a current density of 60 mA g^−1^. (**d**) Rate capability of NP-VO_2_ and NP-VO_2_/rGO at various current densities. The coulombic efficiency of VO_2_/rGO is also shown in (**c,d**).

The electrochemical properties of the NP-VO_2_ and NP-VO_2_/rGO electrodes were evaluated for LIB application in the voltage range of 0.1–3.0 V (vs. Li/Li^+^). Figure 4a shows the cyclic voltammograms of NP-VO_2_ at a scan rate of 0.3 mV s^−1^ up to the 10^th^ cycle, to identify the electrochemical reactions. A well-defined cathodic peak near 1.7 V observed at 1^st^ cathodic scan disappeared in the following scan. According to the TiO_2_ which exhibits the similar crystal structure with VO_2_(M), this irreversible peak near 1.7 V is measured due to the lithium surface storage or the surface defects, vacancies and adsorbed water in nano-sized materials^22^,^23^,^24^. The other irreversible peak between 0.1 and 0.5 V is attributed to unintended side reactions, or to the formation of a solid electrolyte interface (SEI) layer, and/or electrolyte decomposition^25^. During the 2^nd^ scan, four pairs of redox peaks appear, i.e. 2.35/2.76 (a/a′), 1.85/2.10 (b/b′), 0.95/1.20 (c/c′), and 0.50/0.68 V (d/d′). Each redox peak is relevant to different insertion sites. Interestingly, after the 2^nd^ cycle, the separation of the redox peaks between a and a′ became smaller, i.e., the anodic peak (a′) shifted to a lower potential, indicating the improvement in stability and reversibility for the insertion/desertion of the Li ion, as the number of test cycles increased^26^, which exhibited the same phenomenon in CV of NP-VO_2_/rGO (see Supplementary Fig. S3). The redox peaks of b and b′ continuously decreased with an increase in test cycles, while there were significant changes in two redox peaks (c/c′ and d/d′). Meanwhile, large redox peaks of b/b′ near 2 V were measured in NP-VO_2_/rGO at initial scan, but gradually decreased with increasing scan number becoming similar to CV of NP-VO_2_.

Figure 4b shows the galvanostatic charge–discharge profiles of NP-VO_2_ at a current density of 60 mA g^−1^ up to the 200^th^ cycle. The high discharge capacity at the 1^st^ cycle was caused by the aforementioned reasons, namely, unintended side reactions, or the formation of a solid SEI layer, and/or electrolyte decomposition (see Supplementary Fig. S4)^25^. After the 2^nd^ cycle, the plateaus observed in the voltage-specific capacity profiles were in good agreement with the redox peaks in the CV. At the 2^nd^ cycle, the charge and discharge capacities were estimated at 319 and 350 mA h g^−1^, respectively, and the discharge capacity gradually decreased to 281 mA h g^−1^ at the 50^th^ cycle, and 238 mA h g^−1^ at the 100^th^ cycle. However, these capacities are comparable to those of the pristine VO_2_(B) nanoribbons cycled at the same voltage ranges^26^. After the 100^th^ cycle, the degradation of the specific capacity of NP-VO_2_ was mitigated. The discharge capacity at the 200^th^ cycle was measured to 236 mA h g^−1^, corresponding to the capacity retention of more than 99% compared to that at the 100^th^ cycle. Galvanostatic charge-discharge profiles of NP-VO_2_/rGO exhibited the similar tendency with those of NP-VO_2_ (See Supplementary Fig. S5).

Figure 4c shows the comparison between the specific capacities for the NP-VO_2_ and NP-VO_2_/rGO electrodes over 200 charge-discharge cycles at a current density of 60 mA g^−1^. Compared with NP-VO_2_, NP-VO_2_/rGO features a slightly lower initial specific capacity of 267 and 269 mA g^−1^ at the 2^nd^ charge and discharge cycling, respectively. Interestingly however, the discharge capacity increased to 311 mA h g^−1^ at the 10^th^ cycle, due to the possible activation process such as size, good dispersion, or compact contact between the active materials and supports in the working electrode. This resulted in the improvement of the specific capacity during the initial cycling states^27^. After the 10^th^ cycle, NP-VO_2_/rGO exhibited excellent capacity stability, better than that of NP-VO_2_. The discharge capacity of NP-VO_2_/rGO was estimated to 298, 295, and 283 mA h g^−1^ at the 50^th^, 100^th^, and 200^th^ cycle, respectively. This corresponds to capacity retention of approximately 96, 95, and 91%, respectively, compared to the discharge capacity at the 10^th^ cycle. Also, the coulombic efficiency of VO_2_/rGO increases to more than 99% after the initial several cycles, indicating a good reversibility of the reaction of Li ions with VO_2_(M).

The rate capability of NP-VO_2_ and NP-VO_2_/rGO was measured at various current densities (Fig. 4d). The discharge capacities measured for the NP-VO_2_ were estimated to 341, 328, 303, 291, 263, 245 and 221 mA h g^−1^ at a current densities of 20, 60, 80, 100, 150, 200 and 300 mA g^−1^, respectively, indicating good rate capability even at higher current density. In the case of NP-VO_2_/rGO, the initial specific capacities of NP-VO_2_/rGO between 1^st^ and 20^th^ cycle are lower than those of NP-VO_2_ at low current densities (20, 60, 80, 100 mA g^−1^). However, it is considered that this result is not because NP-VO_2_/rGO exhibits inferior rate capability to NP-VO_2_ at low current densities. As aforementioned in Fig. 4c, NP-VO_2_/rGO features a slightly lower initial specific capacities than NP-VO_2_ up to 30^th^ cycle. This phenomenon seemed to be reflected in rate performance. Compared with NP-VO_2_, however, capacity drop in NP-VO_2_/rGO when increasing the current density was relatively lower than that in NP-VO_2_. The discharge capacities of NP-VO_2_/rGO were estimated to 300, 275, 259, 248, 232, 241, 221 mA h g^−1^. Capacity decrease ratio from 20 mA g^−1^ to 300 mA g^−1^ is 26% in NP-VO_2_/rGO, while 35% in NP-VO_2_, indicating higher rate capability of NP-VO_2_/rGO compared to NP-VO_2_.

**Figure 5:**
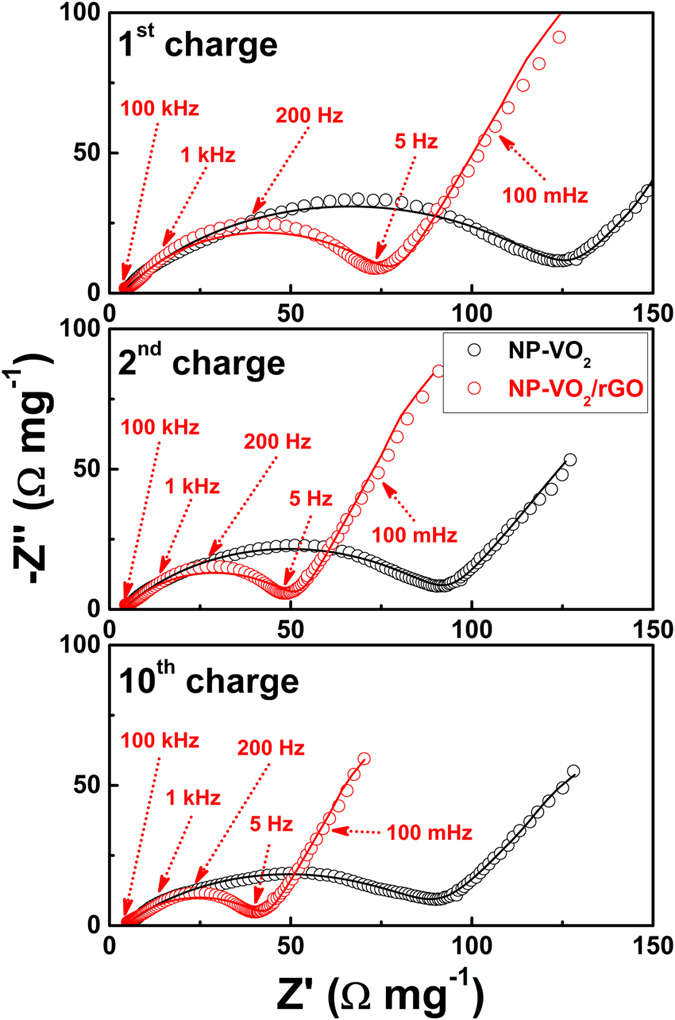
Electrochemical impedance. Nyquist plots for NP-VO_2_ and NP-VO_2_/rGO at the 1^st^, 2^nd^ and 10^th^ charged states. The symbols (open circles) and continuous lines represent the experimental spectra and fit to the data using the equivalent electrical circuit shown in the inset, respectively.

Electrochemical impedance spectroscopy (EIS) was performed at a frequency ranging between 100 kHz and 10 mHz to compare the charge transfer resistances of NP-VO_2_ and NP-VO_2_/rGO (Fig. 5). When measuring the electrochemical impedance, loading weight in both electrodes were almost similar; 1.64 and 1.61 mg for NP-VO_2_ and NP-VO_2_/rGO, respectively. The measured EIS spectra were fitted to the equivalent circuit depicted in the inset of Fig. 5. All the plots show a semicircle in the high-frequency region, corresponding to the charge transfer resistance at the electrode/electrolyte interface. The fitted mean values and their errors of total ohmic resistance for the electrolyte and electrical contacts (Re) and charge transfer resistance (RCT) for the NP-VO_2_ and NP-VO_2_/rGO were presented in Table S1. The lower charge transfer resistance enables a more rapid charge transfer, resulting in faster kinetics of the faradic reaction^28^. Shown in Fig. 5, NP-VO_2_/rGO exhibited a smaller diameter of the high-frequency semicircle than that of NP-VO_2_ at the 1^st^, 2^nd^ and 10^th^ charged states. Also, the fitted R_CT_ values of NP-VO_2_/rGO were 75.51, 49.12, and 40 Ω at 1^st^, 2^nd^ and 10^th^ charged state, respectively, which are lower than those of NP-VO_2_ in all charged states (125.26, 90.72, and 89.63 Ω at 1^st^, 2^nd^ and 10^th^ charged state, respectively), indicating that NP-VO_2_/rGO has a lower charge transfer resistance compared to NP-VO_2_.

## Discussion

In the case of the synthesis of VO_2_ without rGO, the equation for VO_2_ formation may be formulated as follows^15^:


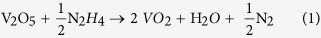


As aforementioned, the amount of N_2_H_4_ was the critical factor in synthesis of NP-VO_2_. When adding a stoichiometric amount of N_2_H_4_ (molar ratio of N_2_H_4_ to V_2_O_5_ is 0.5), reflected peaks in the XRD pattern (see Supplementary Fig. S6) corresponded to the tetragonal unit cell VO_2_(A) with a P4_2_/ncm space group (JCPDS No. 42-0876). Therefore, the equation for VO_2_ formation may be formulated as follows:


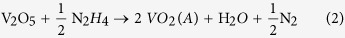


In order to prepare NP-VO_2_, an excess amount of N_2_H_4_ (totally 0.5 + α) was needed.


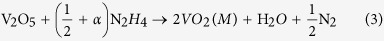


where α ≈ 0.125 in this work. So, excess amount of N_2_H_4_ would force the formation of M-phase VO_2_ in this reaction. If adding more than N_2_H_4_ reductant up to 0.9, M-phase VO_2_ was still synthesized. However, the molar ratio of N_2_H_4_ to V_2_O_5_ was more than 1, phase of the final product was V_2_O_3_ (see Supplementary Fig. S6). That is, it was confirmed that the amount of N_2_H_4_ reductant during a hydrothermal reaction had an influence on the phase of the final product. Meanwhile, when synthesizing NP-VO_2_/rGO, if the molar ratio of GO to V_2_O_5_ gel was β, phase-pure and M-phase VO_2_-rGO could be synthesized by putting additional β/4 mol of N_2_H_4_ (totally 1/2 + α + β/4 mol of N_2_H_4_) in GO-dispersed V_2_O_5_ gel.


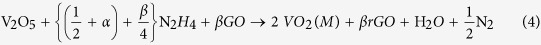


As an additional reductant was lower than β/4 when synthesizing NP-VO_2_/rGO, both VO_2_(A) and VO_2_(M) were detected in the XRD pattern (see Supplementary Fig. S7), indicating that the amount of reductant by which V_2_O_5_ was transformed to the M-phase VO_2_ was insufficient. It is considered that N_2_H_4_ was first used to reduce GO, while the rest of the N_2_H_4_ was used to reduce the V_2_O_5_ gel.

In view of electrochemical information, there have been no reports on Li ion behavior in this VO_2_(M) structure during charging/discharging. In the case of rutile TiO_2_, having a similar crystal structure to VO_2_(M), it is commonly known that the Li ions rapidly diffuse in the TiO_2_ rutile structure along the c-axis channels^29^. Similarly, VO_2_(M) with a distorted rutile structure, exhibits the equivalent channels along the a-axis (see Supplementary Fig. S8). Therefore, it is supposed that Li ions could be intercalated in the VO_2_(M) structure along the direction of the a-axis. However, it is necessary for Li ion migration in the VO_2_(M) structure to be investigated by theoretical calculations.

It is known that rGO could react with Li ions in the voltage range from 0.1 to 3.0 V. Actually, rGO synthesized in this work exhibited the discharge capacity of 250 mA h g^−1^ at 25^th^ cycle (see Supplementary Fig. S9). Considering this, we calculated the discharge capacity of “NP-VO_2_ + rGO” mixture by estimating the capacity contributions of each NP-VO_2_ and rGO from the VO_2_-to-rGO weight ratio. Shown in Fig. S10, the discharge capacity obtained for the NP-VO_2_/rGO is higher than the one estimated based on the simple calculation from the mixture of NP-VO_2_ and rGO, indicating the favorable interaction between NP-VO_2_ and rGO that activate the electrochemical reaction of NP-VO_2_ with Li ions through rGO^30^. In other words, it is confirmed that rGO in VO_2_ NPs has a positive effect on the electrochemical performance of rate capability as well as cyclic retention. The existence of rGO in VO_2_ particles provides an efficient pathway for charge transfer between VO_2_ and the current collector, resulting in a higher reversible specific capacity and rate capability.

In summary, phase-pure and M-phase NP-VO_2_ and NP-VO_2_/rGO were successfully synthesized via a sol-gel assisted hydrothermal process, by controlling the amount of N_2_H_4_ reductant. Each particle size was under 50 nm with or without the existence of GO. The electrochemical properties of this composite were also investigated. VO_2_(M) NPs could be reversibly reacted with Li ions in the voltage range between 0.1 and 3.0 V. The discharge capacity of NP-VO_2_ was 236 mA h g^−1^ at a current density of 60 mA g^−1^ over the 200^th^ cycle. Furthermore, NP-VO_2_/rGO exhibited a higher specific capacity (283 mA h g^−1^ over the 200^th^ cycle) and more cycling stability than NP-VO_2_, as a result of the high electrical conductivity of rGO. Therefore, it is suggested that nanoscale VO_2_(M) could show long-term reversible cycling stability, as well as comparable high specific capacity. Also, through various improvements such as uniform carbon layer coating, micro-structural (doping), or nanostructurized (sheets, rods) changes, this polymorph has a lot of potential for development in LIB application.

## Method

### Materials synthesis

GO solution (2 wt%) was purchased from Angstron Materials. NP-VO_2_/rGO was synthesized via a one-step sol-gel assisted hydrothermal process. GO solution (8 g) was dispersed in a solution of 17 mL deionized water and 5 mL hydrogen peroxide (H_2_O_2_; 30 wt%, OCI Company). Then, 0.9 g V_2_O_5_ (99%, Sigma-Aldrich) powder was dissolved in this solution (total 30 mL) under continuous stirring. After aging for 24 h, the GO-dispersed V_2_O_5_ solution turned into an amorphous gelatinous form. In order to reduce GO-dispersed V_2_O_5_ gel, an appropriate hydrazine monohydrate (N_2_H_4_∙H_2_O; 98%, Sigma-Aldrich) was added to this gel under vigorous mixing. Within a few minutes, the gel turned to a greenish-black-colored stiff gel. The resulting gel was transferred to a 75 mL Teflon-lined stainless steel autoclave, and hydrothermally reacted at 220 °C for 24 h in an electronic oven. After the hydrothermal reaction was complete, the obtained product was collected by centrifugation, thoroughly washed with deionized water and ethanol, and dried in a vacuum oven at 70 °C. To synthesize NP-VO_2_ without rGO (or rGO without NP-VO_2_), V_2_O_5_ powder was dissolved in a solution of 25 mL deionized water and 5 mL H_2_O_2_ (or GO solution was dispersed in a solution of 17 mL deionized water and 5 mL H_2_O_2_), and then added to appropriate N_2_H_4_. The resulting mixture was hydrothermally reacted, centrifuged, washed, and dried as described in the synthesis of VO_2_ NP/rGO, above.

### Materials characterization

X-ray diffraction (XRD) measurements of the powder samples were taken with a Bruker D8-Advance using Cu Kα radiation in the 2θ range of 20 to 70°. Field-emission scanning electron microscopy (FESEM) images were taken by a Hitachi SU-70. High-resolution transmission electron microscopy (HRTEM) analysis, with energy-dispersive spectrometer (EDS) mapping, was conducted by a JEOL JEM-2100F. X-ray photoelectron spectroscopy (XPS) was performed with a Thermo Scientific Sigma Probe, using an Al Kα X-ray source. The amount of rGO content was measured by thermogravimetric analysis (TGA; DTG-60H, Shimadzu Co., Japan) under air atmosphere. The sample was heated at temperatures ranging from room temperature to 600 °C at 10 °C/min under air atmosphere. The three-dimensional visualization crystal structure of VO_2_(M) was illustrated using the VESTA program.

### Electrochemical measurements

The electrochemical properties of NP-VO_2_ and NP-VO_2_/rGO were evaluated using Swagelok-type cells that were fabricated in an Ar-filled glove box. The working electrodes were prepared with 75 wt% active materials, 15 wt% Super P carbon black (MMM Carbon, Belgium), and 15 wt% PVDF-HFP binder (Kynar 2801), and were cast onto Cu foil. The cells were assembled with a working electrode, Li-metal foil as the counter electrode, and a separator film (Celgard 2400) saturated with a liquid electrolyte consisting of 1 M LiPF_6_ dissolved in a solution of ethylene carbonate (EC) and dimethyl carbonate (DMC), at a volume ratio of 1:1. These cells were galvanostatically cycled at voltages ranging from 0.1 to 3.0 V vs. Li/Li^+^, using an automatic battery cycler (WBCS3000, WonATech, Korea). Cyclic voltammetry (CV) measurements were taken in the same voltage range, at a scan rate of 0.3 mV s^−1^. Electrochemical impedance spectroscopy (EIS) measurements were conducted at a frequency range of 100 kHz to 10 mHz with AC amplitude of 10 mV, on an Ivium-n-Stat (Ivium Technologies, Netherlands) electrochemical test system.

## Additional Information

**How to cite this article**: Song, H. J. *et al*. Enhanced Lithium Storage in Reduced Graphene Oxide-supported M-phase Vanadium(IV) Dioxide Nanoparticles. *Sci. Rep.* **6**, 30202; doi: 10.1038/srep30202 (2016).

## Supplementary Material

Supplementary Information
