# Supplementary material for: Enhanced Lithium Storage in Reduced Graphene Oxide-supported M-phase Vanadium(IV) Dioxide Nanoparticles
Source: Sci Rep. 2016 Jul 22;6:30202. doi: 10.1038/srep30202 (PMC4957147; doi:10.1038/srep30202)
Supplement: Supplementary Information [file srep30202-s1.pdf]

Supplementary Information

Enhanced Lithium Storage in Reduced Graphene Oxide-supported M-phase Vanadium(IV) Dioxide Nanoparticles

Hee Jo Song[1], Mingu Choi[2], Jae-Chan Kim[2], Sangbaek Park[1], Chan Woo Lee[1], Seong-Hyeon Hong[1], Byung-Kook Kim[3], and Dong-Wan Kim*[2]

[1] Department of Materials Science and Engineering, Seoul National University, Seoul 151-744, Republic of Korea

[2] School of Civil, Environmental and Architectural Engineering, Korea University, Seoul 136-713, Republic of Korea

[3] High-Temperature Energy Materials Research Center, Korea Institute of Science and Technology, Seoul 136-791, Republic of Korea

* Corresponding author

Prof. Dong-Wan Kim (D.–W. Kim)

Tel: +82-2-3290-4863

E-mail: dwkim1@korea.ac.kr

Table S1. Fitted values of the Nyquist plot for NP-VO_2 and NP-VO_2/rGO.

	NP-VO_2		NP-VO_2/rGO	
	R_e (Ω mg^{-1})	R_{CT} (Ω mg^{-1})	R_e (Ω mg^{-1})	R_{CT} (Ω mg^{-1})
1st	3.32 ±0.16	125.26 ±1.96	4.29 ±0.25	75.51 ±0.24
2nd	3.82 ±0.13	90.72 ±1.09	4.24 ±2.98	49.12 ±1.85
10th	3.97 ±0.28	89.63 ±1.66	4.54 ±0.18	40 ±1.21

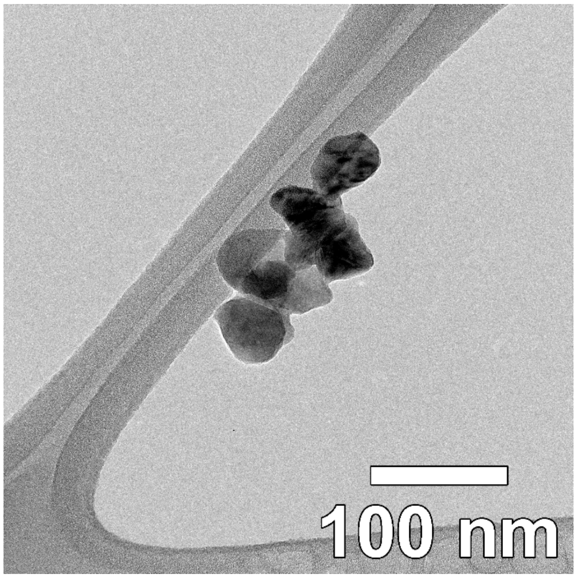

Figure S1. TEM image of NP-VO_2.

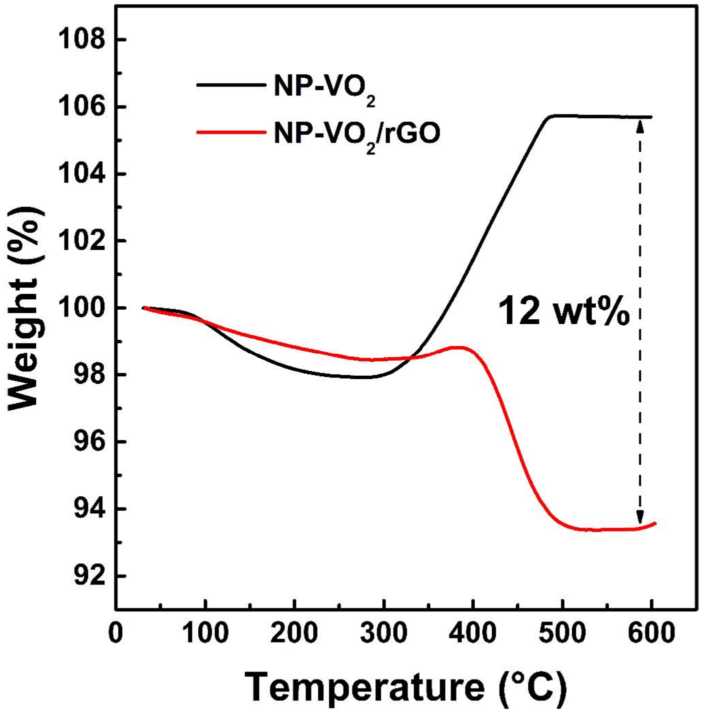

Figure S2. TGA curves of NP-VO_2 and NP-VO_2/rGO.

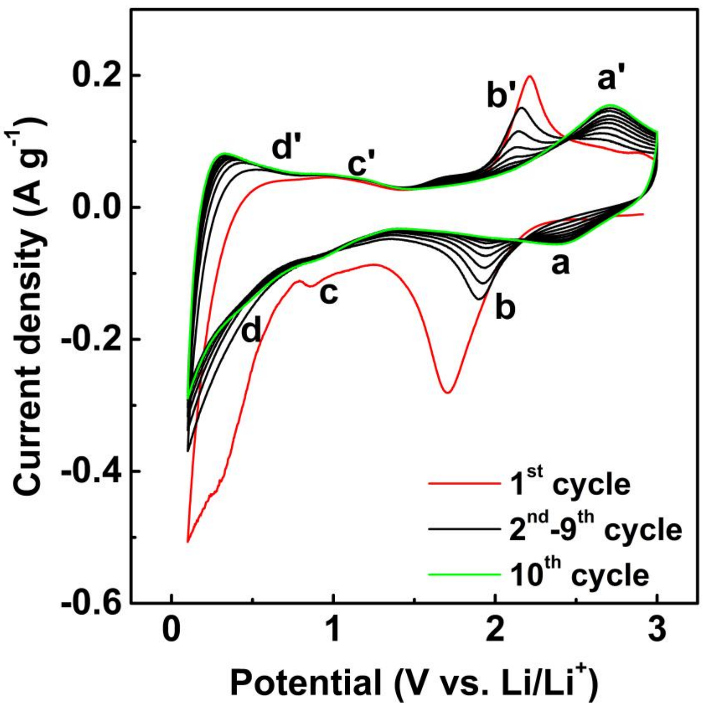

Figure S3. Cyclic voltammetry of NP-VO_2/rGO at a scan rate of 0.3 mV s^{-1} up to the 10th cycle.

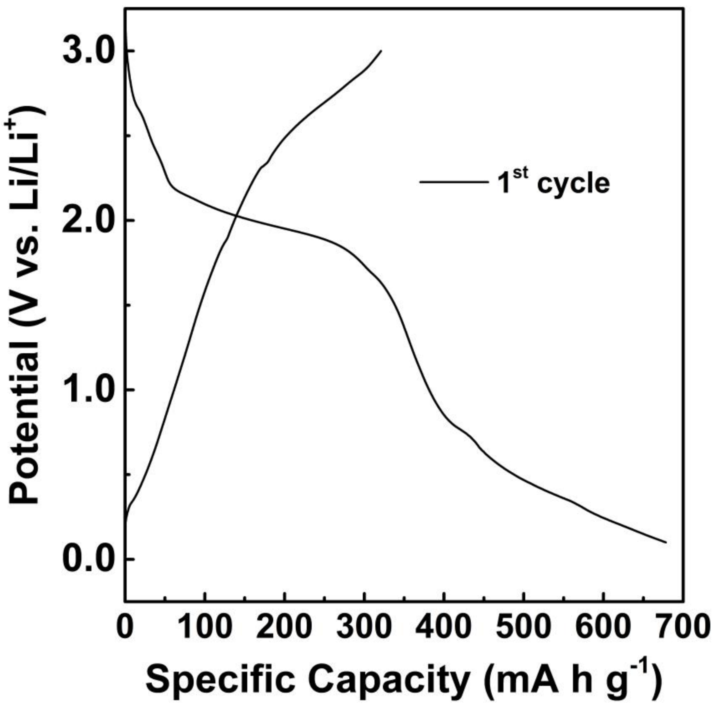

Figure S4. Galvanostatic voltage-specific capacity profiles of NP-VO_2 at a scan rate of 60 mA g^{-1} at the 1st cycle.

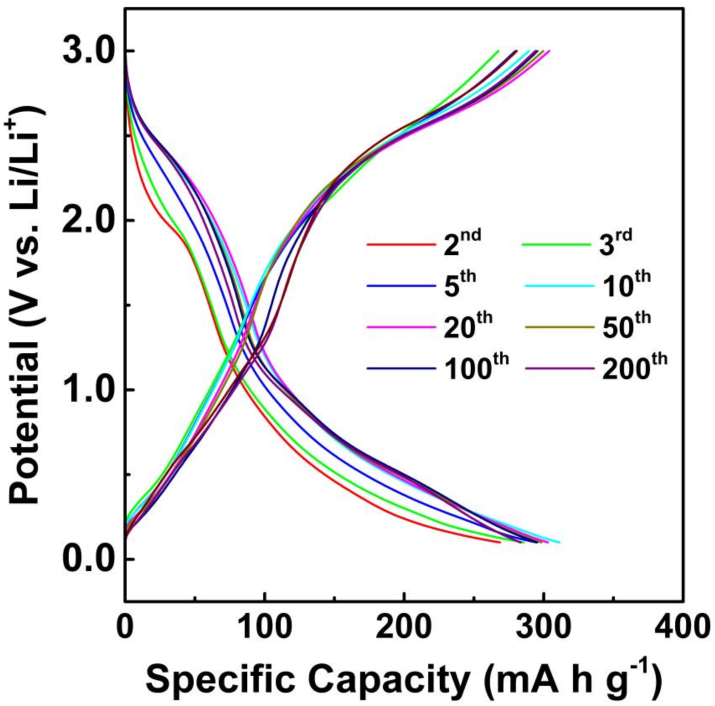

Figure S5. Galvanostatic voltage-specific capacity profiles of NP-VO_2/rGO at a scan rate of 60 mA g^{-1} up to 200th cycle.

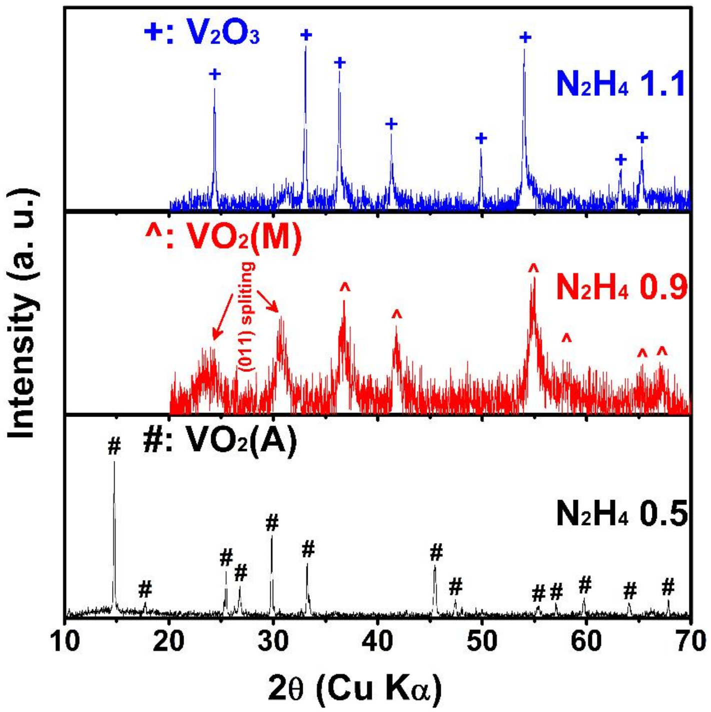

Figure S6. XRD patterns of VO_2 with adding various amount of N_2H_4 reductant.

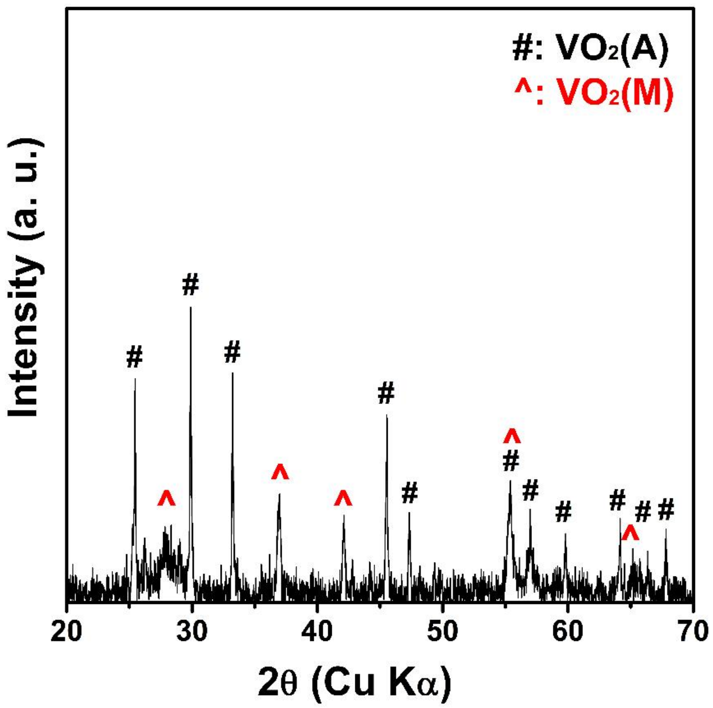

Figure S7. XRD patterns of M- and A-mixed phase VO_2.

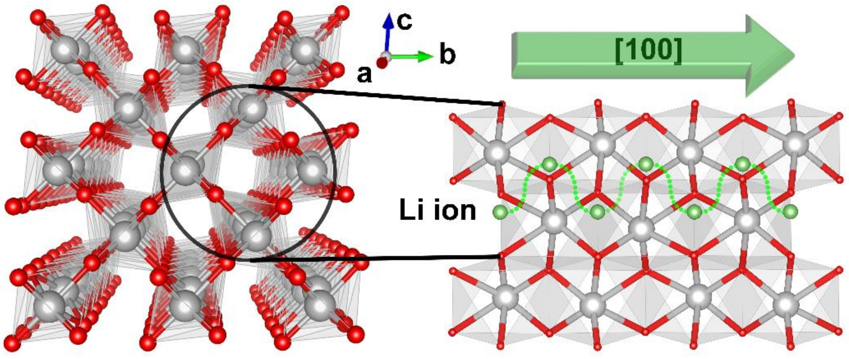

Figure S8. Visualization of Li ion migration in crystal structure of VO_2(M).

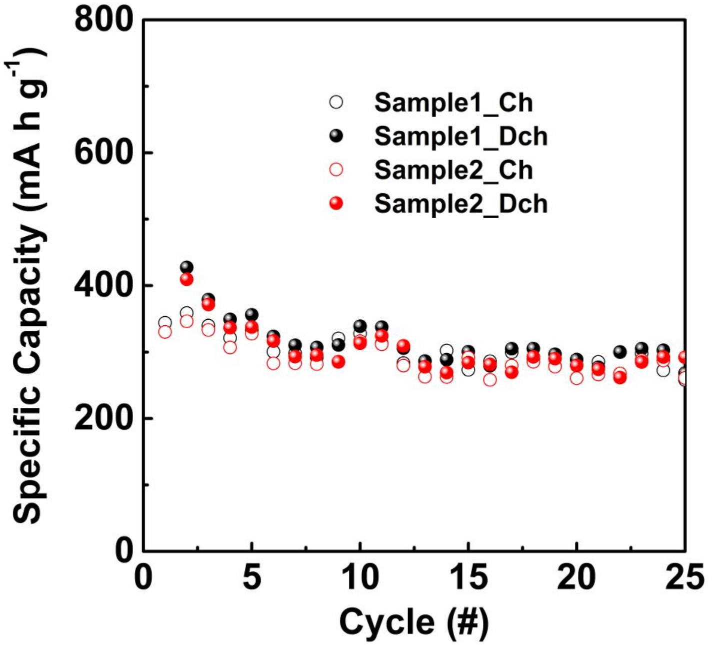

Figure S9. Specific capacities of rGO at a current density of 60 mA g^{-1}.

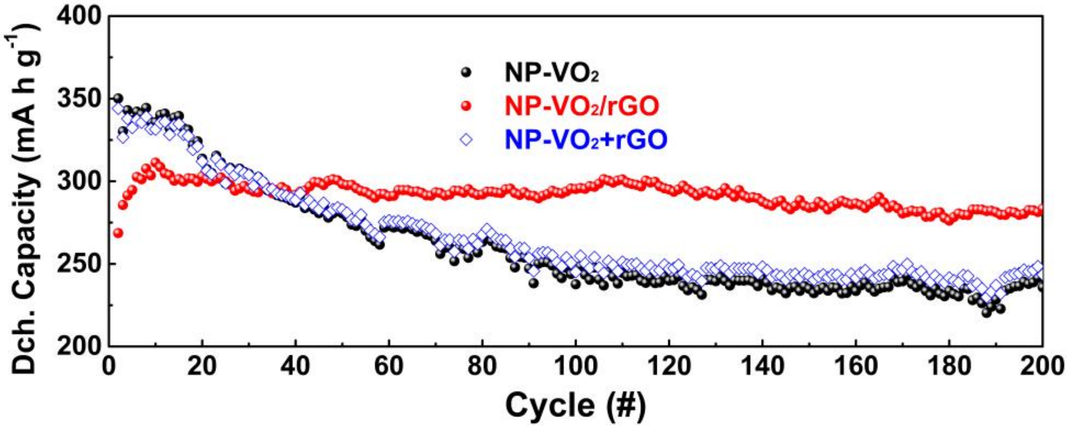

Figure S10. Discharge capacities of NP-VO_2, NP-VO_2/rGO composite and "NP-VO_2 + rGO" mixture.
